# Advancements in the Treatment of Necrotizing Otitis Externa with Hyperbaric Oxygen: A Systematic Review

**DOI:** 10.1055/s-0045-1809432

**Published:** 2025-09-10

**Authors:** Hassan Al Bazzal, Firas Hassan, Mohamad Tlais, Yehya Tlaiss

**Affiliations:** 1 Department of Otolaryngology, Al Rayyan Hospital, Lebanon; 2 Department of Otolaryngology, Faculty of Medicine and Medical Sciences, University of Balamand, Lebanon

**Keywords:** hyperbaric oxygenation, otitis externa, necrotizing diabetes mellitus, adjuvant therapy

## Abstract

**Introduction:**

Necrotizing otitis externa (NOE), also known as *malignant otitis externa* (MOE), is a severe infection that begins in the external auditory canal and can extend to adjacent tissues and bone. It primarily affects elderly, diabetic, and immuno-compromised patients. Despite the advancements in antibiotics and surgical interventions, NOE remains a condition with significant morbidity and mortality.

**Objective:**

To evaluate the efficacy of hyperbaric oxygen therapy (HBOT) as an adjunctive treatment for NOE, focusing on clinical outcomes and the potential benefits in refractory or advanced cases.

**Methods:**

We conducted a comprehensive literature search on the PubMed/MEDLINE and Cochrane Library databases for articles published from January 1980 to December 2023. The search terms included *hyperbaric oxygen therapy* , *necrotizing otitis externa* , and *refractory otitis* . A total of 8 studies met the inclusion criteria, comprising case reports, observational studies, and case series. Study quality was assessed through the Cochrane Risk of Bias tool and the Newcastle-Ottawa Scale.

**Results:**

The results indicate that HBOT shows promise in the treatment of NOE, with several studies reporting complete resolution of infection and prevention of recurrence, especially in refractory cases.

**Conclusion:**

The current evidence is insufficient to establish HBOT as a standard treatment for NOE; however, its potential benefits in improving clinical outcomes and reducing morbidity are significant. High-quality research, including randomized controlled trials, is necessary to validate the role of HBOT in NOE treatment. Where hyperbaric facilities are accessible, HBOT should be considered for refractory NOE cases.

## Introduction

Necrotizing otitis externa (NOE) is a severe infection that starts in the external auditory canal and can spread to surrounding tissue and, eventually, bone. 1 It is potentially fatal and, historically, it has a high mortality rate. 2 Though called *malignant otitis externa* (MOE) in the older literature, it is non-cancerous. 3 Hence, the term *necrotizing otitis externa* (NOE) is more accurate, reflecting the severe and rapidly-spreading nature of the disease. 4 This condition predominantly affects elderly individuals, diabetes patients, and immunocompromised subjects. 5 It is mostly caused by the bacteria *Pseudomonas aeruginosa* ; nonetheless, other bacteria and fungi have also been reported. 6 7 8 Accordingly, topical and systematic antibiotics or antifungals are used in the treatment, as well as surgical debridement of necrotic tissue in the external auditory canal and surrounding structures in case of extensive disease. 9 10 Despite the advancements in medications and surgical management, NOE remains a serious illness associated with considerable morbidity and mortality. 11 12

Hyperbaric oxygen therapy (HBOT) has emerged as a potential adjunctive therapy for NOE in cases of refractory or advanced disease; 13 14 it involves intermittent administration of 100% oxygen, while the patient is inside a compression chamber, followed by the application of pressures greater than the atmospheric pressure. 15 Although the American Undersea and Hyperbaric Medical Society (UHMS) recommends the use of HBOT in cases of refractory osteomyelitis and necrotizing inflammation, it is not considered a definitive treatment in NOE due to shortage of evidence in the literature. 16 The present systematic review aims to investigate and evaluate the latest evidence on the efficacy of HBOT as an adjunctive therapy for NOE management.

## Literature Review

### Pathophysiology of NOE

The most common etiology of NOE is *P. aeruginosa,* a gram-negative bacillus. 17 Additionally, gram-negative bacteria, such as *Klebsiella* and *Proteus* spp., have also been reported. 18 19 Fungal infections, especially by *Candida* and *Aspergillus* spp., can cause NOE as well. 20 21

This infection is most common in elderly, diabetic, and immunocompromised patients, such as those with HIV or those undergoing chemotherapy. 5 17 Two factors make diabetic patients more susceptible to external otitis: poorly-controlled diabetes can induce immune dysfunction and small-vessel vasculopathy; and the external ear cerumen (earwax) of diabetic patients presents a more basic pH and decreased concentration of lysosomes, making the external auditory canal more prone to infection. 1 Immunocompromise not related to diabetes can be caused by HIV and chemotherapy, for example: these patients tend to develop the infection at a younger age compared to diabetic subjects, 22 and they tend to have a worse prognosis. 23

Typically, NOE starts as a simple soft-tissue infection of the auricle and external auditory canal (EAC). 1 It can then progress from cellulitis to chondritis, periostitis, and, eventually, osteomyelitis. 24 The pathway of the infection extends to the osseocartilaginous junction of the EAC through the fissures of Santorini until it reaches the dural sinuses and petrous apex via the fascial and vascular planes, 25 resulting in bony erosion and invasion of collateral tissues until structures such as the skull base and cranial nerves are involved. 26 Hence, inflammation in areas such as the hypoglossal canals and the stylomastoid and jugular foramina, along with all their structures, is known to occur. 27

The patients mainly present with symptoms of deep ear pain that gets worse with motion, ear discharge, and hearing loss. 28 Due to deeper tissue invasions, symptoms may also manifest as palsies of the following cranial nerves: facial (VII), glossopharyngeal (IX), vagus (X), accessory (XI), and hypoglossal (XII). 29 Subsequently, patients may also present with hoarseness, shoulder weakness, dysphagia, and facial and tongue weakness. 27

Additionally, the limited blood supply to cartilage, which is further exacerbated by the vasculopathy of diabetes, presents a significant challenge in the treatment of NOE. 25 Even parenteral antibiotics are slow to achieve bactericidal levels in cartilage. 25 Hyperbaric oxygen therapy helps oxygenate the tissues, and natural immune processes work better when the necessary cells have sufficient oxygen.

### Rationale for HBOT

The primary treatment for NOE involves long-term topical and systemic antibiotics (for 4–6 weeks) in addition to careful monitoring of blood glucose levels. 30 31 If the infection is of fungal origin, then, intensive antifungals are required. 32 In severe cases or when primary treatment is ineffective, surgical debridement of necrotic tissue or abscess drainage may also be required. 33 Even though new antimicrobials are now used, resistant strains might emerge, and the disease can enter a refractory stage when surgical and non-surgical treatments are ineffective. 34 As previously mentioned, diabetes is highly prevalent in NOE: more than 80% of NOE patients suffer from diabetes. 35 The diabetic effects of microangiopathy-induced hypoxia and leukocyte immunosuppression predispose to NOE exacerbations. 36 The hypoxia and hypoperfusion caused by diabetic microangiopathy inhibit the oxygen-dependent antimicrobial activity of leukocytes, while the infection, in turn, consumes most of the oxygen in the tissue via bacterial absorption and inflammatory processes. 37 38 Since oxygen deficiency in tissues is what leads to necrosis and quick expansion of the NOE infection, exposure to hyperbaric oxygen could be used to counter these effects by increasing the levels of oxygen in the tissue and promoting its healing and angiogenesis. Hence, HBOT has been proposed as a potential adjunctive therapy due to its ability to enhance tissue oxygenation, promote capillary angiogenesis, and exert antimicrobial effects. 39 40 Moreover, clinical success has been reported 43 44 regarding HBOT in the adjunctive treatment of chronic or refractory osteomyelitis.

## Methods

### Search Strategy

We conducted a comprehensive literature search in the PubMed/MEDLINE and Cochrane Library databases for articles published from January 1980 to December 2023. The search terms included *hyperbaric oxygen therapy* , *malignant otitis externa* , *necrotizing otitis externa* , and *refractory otitis* . We selected studies published in English on the use of HBOT in NOE, including case reports, observational studies, and clinical trials.

### Study Selection

**Fig. 1:**
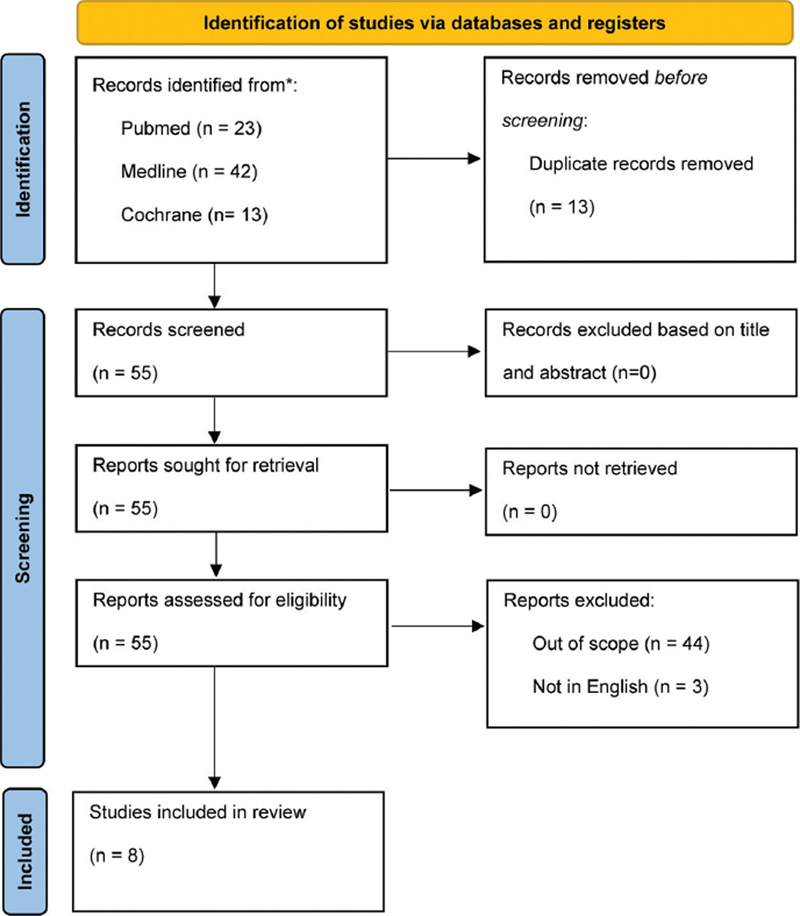
Preferred Reporting Items for Systematic Reviews and Meta-Analyses (PRISMA) flow diagram of the study selection process.

The Inclusion criterium for article selection was studies on HBOT as an adjunctive therapy for NOE, and the exclusion criteria were studies not involving HBOT, reviews, and editorials. Two independent reviewers screened the titles and abstracts of all identified studies and then reviewed the full texts of potentially relevant studies. Disagreements were resolved through discussion and consensus. A total of 78 studies were identified through the database search. After removing duplicates and screening titles and abstracts, 55 full-text articles were assessed for eligibility; 8 studies, 40 41 42 43 44 45 48 49 which included case reports, observational studies, and case series, met the inclusion criterium ( Fig. 1 ).

### Data Extraction

Data were extracted using a standardized form that included study characteristics (author, year, study design), patient characteristics (age, comorbidities), intervention details (HBOT protocol), and outcomes (infection resolution, recurrence, adverse effects).

### Quality Assessment

The quality of the included studies was assessed through the Cochrane Risk of Bias tool for randomized controlled trials (RoB 1, The Cochrane Collaboration, London, United Kingdom) and the Newcastle-Ottawa Scale (NOS) for observational studies. Each study was evaluated in terms of bias in selection, performance, detection, attrition, and reporting. The quality assessment revealed a range of methodological limitations across the studies, including small sample sizes, lack of control groups, and potential selection bias. Most studies were rated as presenting moderate to high risk of bias.

## Discussion

Early data on HBOT adjunctive therapy for NOE treatment were conflicting. The first case was reported as early as the 1980s, when Mader and Love 40 opted for HBOT therapy in a refractory case of NOE in a patient with diabetes undergoing moxalactam disodium therapy. In total 20 sessions took place with the HBOT set at 2.5 atmospheric pressure inside the chamber for 90 minutes. The infection resolved completely, with no recurrence. In contrast, Joachims et al. 41 reported a series of 4 cases of NOE which did not respond to the accepted treatment of long-term systemic antibiotics followed by surgical intervention, with HBOT supplementation in refractory cases; the HBOT setting was similar to that used by Mader and Love, 40 with 40 sessions that were unsuccessful until ciprofloxacin, a newly-developed fluoroquinolone, was administered. This raises suspicion regarding any confounding variables between the adjunctive HBOT, and the class of antibiotics used. Similarly, Gordon and Giddings 42 reported two cases of fungal NOE refractory to HBOT, successful treatment was only achieved when a more potent course of antifungals was administered. Conversely, in a retrospective observational study, Davis et al. 43 evaluated 16 patients successfully treated with HBOT, with no recurrence or adverse effects even after years of follow-up examinations. More recently, with the development of the newest generation of antibiotics and advanced surgical techniques, HBOT adjunctive therapy found much more effective results. Another retrospective study, by Amaro et al., 44 presented 16 patients, 60% of whom had nerve palsies and were in a refractory state; HBOT was performed on an average of 34 sessions, and the result was successful for all patients. However, data on follow-up was not available; hence, information on adverse effects and recurrence could not be collected. Singh et al. 45 performed a retrospective analysis of three cases of inadequately treated NOE in elderly diabetic individuals; two of the three cases died of the disease despite aggressive treatment. One case was treated successfully with a combination of antipseudomonal microbial drugs for 8 to 12 weeks and HBOT. Narozny et al. highlighted the therapeutic benefit of HBOT in both bacterial and fungal NOE cases, suggesting its versatility as an adjunct treatment. 46 Given the aggressive nature of NOE and the importance of timely intervention, accurate diagnosis is essential for guiding therapy, including HBOT. Okpala et al. underscored the value of radionuclide imaging in detecting skull base involvement, which is critical in identifying candidates for adjunctive HBOT. 47 Ling and Sader 48 reported a case of a patient with fungal NOE treated with HBOT who presented complete recovery. The HBOT settings across the included studies were inconsistent, with variations in pressure, duration, and total number of sessions. While some studies clearly described their protocols --such as Mader and Love (20 sessions at 2.5 atm for 90 minutes), 40 Amaro et al. (average of 34 sessions), 44 and Al Siyabi et al. (average of 29 sessions) 49 --others either lacked precise descriptions or did not report the parameters at all, including the studies by Gordon and Giddings, 42 , Davis et al., 43 Singh et al., 45 and Ling and Sader. 48 Finally, in 2023, Al Siyabi et al. 49 reported a case series of 20 patients submitted to an average of 29 HBOT sessions in which 19 were cured.

The synthesis of the results indicates that HBOT, as an adjunctive therapy, showed promise in the treatment of refractory or advanced cases of NOE. Several case reports and observational studies have reported complete resolution of infection and prevention of recurrence with HBOT, including those by Mader and Love, 40 Davis et al., 43 Amaro et al., 44 and Al Siyabi et al. 49 However, the heterogeneity in study designs and HBOT protocols, along with the lack of randomized controlled trials, limits the ability to draw definitive conclusions. ( Table 1 ) summarizes the studies.

**Table 1:** Summary of HBOT studies and their outcomes

Type of study	Author (year)	Description	Outcome
Case report	Mader and Love 40 (1982)	HBOT in refractory case of NOE in a patient with diabetes undergoing moxalactam disodium therapy	The infection resolved completely with no recurrence
Case series	Joachims et al. 41 (1988)	Four cases of NOE which did not respond to the accepted treatment of long-term systemic antibiotics followed by surgical intervention, with HBOT supplementation in refractory cases	No success until ciprofloxacin administration
Case report	Gordon and Giddings 42 (1994)	Two patients with NOE cause by Aspergillus flavus	No success with HBOT; successful treatment with more potent course of antifungals
Observational study	Davis et al. 43 (1992)	Retrospective observational study with 16 patients treated with HBOT	Complete treatment with no recurrence or adverse effects
Observational study	Amaro et al. 44 (2019)	HBOT performed for 16 patients, 60% with nerve palsies and refractory state	Successful treatment for all patients, but no follow-up data available
Case report	Singh et al. 45 (2005)	Retrospective analysis of three cases of inadequately treated NOE in elderly diabetic individuals	Two of the three cases died of the disease despite aggressive treatment. One case was treated successfully with a combination of antipseudomonal microbial drugs for 8 to 12 weeks and HBOT
Case report	Ling and Sader 48 (2008)	One patient with fungal NOE treated with HBOT	Complete recovery
Case series	Al Siyabi et al. 49 (2023)	Twenty patients who underwent an average of 29 sessions of HBOT	19 out of 20 patients were cured

**Abbreviations:** HBOT, hyperbaric oxygen therapy; NOE, necrotizing otitis externa.

Hyperbaric oxygen therapy can improve oxygenation in hypoxic tissues, enhancing immune mechanisms dependent on oxygen. It also has bacteriostatic and bactericidal properties against certain pathogens such as *P. aeruginosa* . 53 In a retrospective analysis of 15 patients, Gomes et al. 53 reported that HBOT led to complete disease remission in all cases after 40 to 60 sessions, with no reported mortality or recurrence during a 1-year follow-up. They 53 highlighted the usefulness of HBOT in the management of refractory NOE, noting its synergy with antibiotics and limited adverse effects. However, prolonged treatment durations (typically 40–60 sessions) and associated costs remain barriers. 53

The findings of the current systematic review suggest that HBOT may be a beneficial adjunctive therapy for NOE, particularly in refractory or advanced cases. The mechanism of action, enhancing tissue oxygenation and promoting healing, aligns with the pathophysiology of NOE. Despite the promising results, the lack of randomized controlled trials and the heterogeneity of existing studies highlight the need for more rigorous research.

## Limitations

Notably, no randomized controlled trials have been conducted on the efficacy of HBOT as an adjunctive treatment for NOE, nor has the efficacy of HBOT been compared with that of the antibiotic and surgical treatments. 30 53 The lack of randomized controlled trials remains a major limitation, as also emphasized by the Cochrane Review by Phillips and Jones. 51 The lack of such evidence could be attributed to the rarity of NOE as well as the poor accessibility to hyperbaric facilities. 35 51

Most of the data herein contained was extracted from retrospective observational studies and case reports in different clinical settings. Thus, much of the evidence may have been under the risk of reporting bias. There is no controlled setting, and the administrators of the hyperbaric chamber may be subject to researcher bias or human errors. This issue becomes more prominent when we assess the fact that in some of the cases mentioned here, 41 42 43 the groups of patients either had a 100% cure rate or a 0% cure rate.

Moreover, it must be considered that older studies may not be as reliable today due to the use of older generations of medications and surgeries which may explain why older studies tended to report fewer positive outcomes, 41 42 especially since more potent antibiotics were still being manufactured at the time and new debridement techniques hadn't yet been developed. This was observed in two studies 40 41 in which the treatment was only successful after the administration of newly manufactured antibiotics and antifungals. Incidentally, there is no data about the difference between fungal and bacterial NOE in the context of HBOT therapy. Aside from the typical side effects of fatigue and lightheadedness, 52 no adverse effects were of note in any of the studies.

## Conclusion

There is no definitive evidence backed by randomized controlled trials about the efficacy of HBOT as an adjunctive therapy for NOE. However, the success of treatment outcomes in recent years makes HBOT highly recommended where facilities are available, especially in refractory cases. Future research should focus on conducting well-designed randomized controlled trials to establish the role of HBOT in NOE treatment.

## References

[1] Al Aaraj M S Kelley C Necrotizing (Malignant) Otitis Externa Treasure Island (FL) StatPearls Publishing; October 29, 2023 32310598

[2] Su N Syed I Garth R Skull based osteomyelitis due to postsurgery malignant otitis externa presenting as stroke BMJ Case Rep 2011 2011 2.20113908E8. Published 2011 May 24 10.1136/bcr.02.2011.3908 PMC3105569 22696765

[3] Dabholkar J P Sheth A Malignant otitis externa Indian J Otolaryngol Head Neck Surg 2001 53 01 55 56 10.1007/BF02910982 23119754 PMC3450864

[4] Hasnaoui M Ben Mabrouk A Chelli J Necrotising otitis externa: A single centre experience J Otol 2021 16 01 22 26 10.1016/j.joto.2020.07.005 33505446 PMC7814081

[5] Treviño González J L Reyes Suárez L L Hernández de León J E Malignant otitis externa: An updated review Am J Otolaryngol 2021 42 02 102894 10.1016/j.amjoto.2020.102894 33429178

[6] Bowles P F Perkins V Schechter E Fungal malignant otitis externa BMJ Case Rep 2017 2017 2.01621842E9. Published 2017 Mar 27 10.1136/bcr-2016-218420 PMC5372270 28348261

[7] Tarazi A E Al-Tawfiq J A Abdi R F Fungal malignant otitis externa: pitfalls, diagnosis, and treatment Otol Neurotol 2012 33 05 769 773 10.1097/MAO.0b013e3182565b46 22664903

[8] Bovo R Benatti A Ciorba A Libanore M Borrelli M Martini A Pseudomonas and Aspergillus interaction in malignant external otitis: risk of treatment failure Acta Otorhinolaryngol Ital 2012 32 06 416 419 23349563 PMC3552534

[9] Mahdyoun P Pulcini C Gahide I Necrotizing otitis externa: a systematic review Otol Neurotol 2013 34 04 620 629 10.1097/MAO.0b013e3182804aee 23598690

[10] Karaman E Yilmaz M Ibrahimov M Haciyev Y Enver O Malignant otitis externa J Craniofac Surg 2012 23 06 1748 1751 10.1097/SCS.0b013e31825e4d9a 23147298

[11] Jacobsen L M Antonelli P J Errors in the diagnosis and management of necrotizing otitis externa Laryngoscope 2010 120 04 S207 10.1002/lary.21673 21225805

[12] Marina S Goutham M K Rajeshwary A Vadisha B Devika T A retrospective review of 14 cases of malignant otitis externa J Otol 2019 14 02 63 66 10.1016/j.joto.2019.01.003 31223303 PMC6570638

[13] Phillips J S Jones S E Hyperbaric oxygen as an adjuvant treatment for malignant otitis externa Cochrane Database Syst Rev 2005 02 CD004617. Published 2005 Apr 18 10.1002/14651858.CD004617.pub2 15846724

[14] Weaver L 13rd ed: Undersea and Hyperbaric Medical Society 2014

[15] Takata J Hopkins M Alexander V Systematic review of the diagnosis and management of necrotising otitis externa: Highlighting the need for high-quality research Clin Otolaryngol 2023 48 03 381 394 10.1111/coa.14041 36759416

[16] Yang T H Kuo S T Young Y H Necrotizing external otitis in a patient caused by Klebsiella pneumoniae Eur Arch Otorhinolaryngol 2006 263 04 344 346 10.1007/s00405-005-0998-y 16378221

[17] Getaneh A Ayalew G Belete D Jemal M Biset S Bacterial Etiologies of Ear Infection and Their Antimicrobial Susceptibility Pattern at the University of Gondar Comprehensive Specialized Hospital, Gondar, Northwest Ethiopia: A Six-Year Retrospective Study Infect Drug Resist 2021 14 4313 4322. Published 2021 Oct 20 10.2147/IDR.S332348 34707376 PMC8542893

[18] Chaudhary H A Ibrahim W H Yousaf Z Abubeker I Y Kartha A Fungal Malignant Otitis Externa Involves a Cascade of Complications Culminating in Pseudoaneurysm of Internal Maxillary Artery: A Case Report Am J Case Rep 2019 20 562 566. Published 2019 Apr 21 10.12659/AJCR.913469 31005959 PMC6489412

[19] Martel J Duclos J Y Darrouzet V Guyot M Bébéar J P Otites externes “malignes” ou nécrosantes progressives[Malignant or necrotizing otitis externa: experience in 22 cases]Ann Otolaryngol Chir Cervicofac 2000 117 05 291 11084403

[20] Walton J Coulson C Fungal malignant otitis externa with facial nerve palsy: tissue biopsy AIDS diagnosis Case Rep Otolaryngol 2014 2014 192318 10.1155/2014/192318 24649388 PMC3933303

[21] Tsilivigkos C Avramidis K Ferekidis E Doupis J Malignant External Otitis: What the Diabetes Specialist Should Know-A Narrative Review Diabetes Ther 2023 14 04 629 638 10.1007/s13300-023-01390-9 36897495 PMC10064349

[22] Lee S K Lee S A Seon S W Analysis of Prognostic Factors in Malignant External Otitis Clin Exp Otorhinolaryngol 2017 10 03 228 235 10.21053/ceo.2016.00612 27671716 PMC5545692

[23] Sreepada G S Kwartler J A Skull base osteomyelitis secondary to malignant otitis externa Curr Opin Otolaryngol Head Neck Surg 2003 11 05 316 323 10.1097/00020840-200310000-00002 14502060

[24] van Kroonenburgh A MJL van der Meer W L Bothof R JP van Tilburg M van Tongeren J Postma A A Advanced Imaging Techniques in Skull Base Osteomyelitis Due to Malignant Otitis Externa Curr Radiol Rep 2018 6 01 3 10.1007/s40134-018-0263-y 29416952 PMC5778178

[25] Singh J Bhardwaj B The Role of Surgical Debridement in Cases of Refractory Malignant Otitis Externa Indian J Otolaryngol Head Neck Surg 2018 70 04 549 554 10.1007/s12070-018-1426-0 30464914 PMC6224839

[26] Mani N Sudhoff H Rajagopal S Moffat D Axon P R Cranial nerve involvement in malignant external otitis: implications for clinical outcome Laryngoscope 2007 117 05 907 910 10.1097/MLG.0b013e318039b30f 17473694

[27] Kaya İ Sezgin B Eraslan S Malignant Otitis Externa: A Retrospective Analysis and Treatment Outcomes Turk Arch Otorhinolaryngol 2018 56 02 106 110 10.5152/tao.2018.3075 30197809 PMC6123115

[28] Resouly A Payne D J Shaw K M Necrotising otitis externa and diabetic control Lancet 1982 1 8275 805 806 10.1016/s0140-6736(82)91857-8 6121256

[29] Mion M Bovo R Marchese-Ragona R Martini A Outcome predictors of treatment effectiveness for fungal malignant external otitis: a systematic review Acta Otorhinolaryngol Ital 2015 35 05 307 313 10.14639/0392-100X-669 26824911 PMC4720925

[30] Krishnamoorthy M Othman N AN Hassan N EB Hitam S B Candida Skull Base Osteomyelitis: a Case Report and Literature Review Acta Medica (Hradec Kralove) 2020 63 02 82 85 10.14712/18059694.2020.22 32771074

[31] Berenholz L Katzenell U Harell M Evolving resistant pseudomonas to ciprofloxacin in malignant otitis externa Laryngoscope 2002 112 09 1619 1622 10.1097/00005537-200209000-00017 12352675

[32] Byun Y J Patel J Nguyen S A Lambert P R Hyperbaric oxygen therapy in malignant otitis externa: A systematic review of the literature World J Otorhinolaryngol Head Neck Surg 2020 7 04 296 302. Published 2020 May 4 10.1016/j.wjorl.2020.04.002 34632343 PMC8486695

[33] Geerlings S E Hoepelman A I Immune dysfunction in patients with diabetes mellitus (DM) FEMS Immunol Med Microbiol 1999 26(3-4):259 265 10.1111/j.1574-695X.1999.tb01397.x 10575137

[34] Knighton D R Halliday B Hunt T K Oxygen as an antibiotic. The effect of inspired oxygen on infection Arch Surg 1984 119 02 199 204 10.1001/archsurg.1984.01390140057010 6365032

[35] Mader J T Brown G L Guckian J C Wells C H Reinarz J A A mechanism for the amelioration by hyperbaric oxygen of experimental staphylococcal osteomyelitis in rabbits J Infect Dis 1980 142 06 915 922 10.1093/infdis/142.6.915 7462700

[36] Hunt T K Pai M P The effect of varying ambient oxygen tensions on wound metabolism and collagen synthesis Surg Gynecol Obstet 1972 135 04 561 567 5077722

[37] Bingham E L Hart G B Hyperbaric oxygen treatment of refractory osteomyelitis Postgrad Med 1977 61 06 70 76 10.1080/00325481.1977.11712216 866286

[38] Savvidou O D Kaspiris A Bolia I K Effectiveness of Hyperbaric Oxygen Therapy for the Management of Chronic Osteomyelitis: A Systematic Review of the Literature Orthopedics 2018 41 04 193 199 10.3928/01477447-20180628-02 30035798

[39] Shields R C Nichols F C Buchta W G Claus P L Hyperbaric oxygen therapy for chronic refractory osteomyelitis of the sternum Ann Thorac Surg 2010 89 05 1661 1663 10.1016/j.athoracsur.2009.10.018 20417809

[40] Mader J T Love J T Malignant external otitis. Cure with adjunctive hyperbaric oxygen therapy Arch Otolaryngol 1982 108 01 38 40 10.1001/archotol.1982.00790490040011 6459078

[41] Joachims H Z Danino J Raz R Malignant external otitis: treatment with fluoroquinolones Am J Otolaryngol 1988 9 03 102 105 10.1016/s0196-0709(88)80014-0 3177762

[42] Gordon G Giddings N A Invasive otitis externa due to Aspergillus species: case report and review Clin Infect Dis 1994 19 05 866 870 10.1093/clinids/19.5.866 7893871

[43] Davis J C Gates G A Lerner C Davis M G Jr Mader J T Dinesman A Adjuvant hyperbaric oxygen in malignant external otitis Arch Otolaryngol Head Neck Surg 1992 118 01 89 93 10.1001/archotol.1992.01880010093022 1728284

[44] Amaro C E Espiney R Radu L Guerreiro F Malignant (necrotizing) externa otitis: the experience of a single hyperbaric centre Eur Arch Otorhinolaryngol 2019 276 07 1881 1887 10.1007/s00405-019-05396-7 31165255

[45] Singh A Al Khabori M Hyder M J Skull base osteomyelitis: diagnostic and therapeutic challenges in atypical presentation Otolaryngol Head Neck Surg 2005 133 01 121 125 10.1016/j.otohns.2005.03.024 16025065

[46] Narozny W Kuczkowski J Stankiewicz C Kot J Mikaszewski B Przewozny T Value of hyperbaric oxygen in bacterial and fungal malignant external otitis treatment Eur Arch Otorhinolaryngol 2006 263 07 680 684 10.1007/s00405-006-0033-y 16633825

[47] Okpala N C Siraj Q H Nilssen E Pringle M Radiological and radionuclide investigation of malignant otitis externa J Laryngol Otol 2005 119 01 71 75 10.1258/0022215053222978 15807974

[48] Ling S S Sader C Fungal malignant otitis externa treated with hyperbaric oxygen Int J Infect Dis 2008 12 05 550 552 10.1016/j.ijid.2008.03.003 18508401

[49] Al Siyabi A Al Farsi B Al-Shidhani A Al Hinai Z Al Balushi Y Al Qartoobi H Management of Malignant Otitis Externa with Hyperbaric Oxygen Therapy: A Case Series of 20 Patients Oman Med J 2023 38 03 e512. Published 2023 May 31 10.5001/omj.2023.19 37325261 PMC10264722

[50] Gomes P M Cabral D C Costa J B Hyperbaric oxygen therapy in malignant otitis externa: a retrospective analysis Eur Arch Otorhinolaryngol 2024 281 10 5153 5157 10.1007/s00405-024-08734-6 38767696

[51] Phillips J S Jones S E Hyperbaric oxygen as an adjuvant treatment for malignant otitis externa Cochrane Database Syst Rev 2013 2013 05 CD004617. Published 2013 May 31 10.1002/14651858.CD004617.pub3 23728650 PMC7389256

[52] Saxby A Barakate M Kertesz T James J Bennett M Malignant otitis externa: experience with hyperbaric oxygen therapy Diving Hyperb Med 2010 40 04 195 200 23111934

[53] Heyboer M III Sharma D Santiago W McCulloch N Hyperbaric Oxygen Therapy: Side Effects Defined and Quantified Adv Wound Care (New Rochelle) 2017 6 06 210 224 10.1089/wound.2016.0718 28616361 PMC5467109

